# Valley-selective optical Stark effect of exciton-polaritons in a monolayer semiconductor

**DOI:** 10.1038/s41467-021-24764-8

**Published:** 2021-07-26

**Authors:** Trevor LaMountain, Jovan Nelson, Erik J. Lenferink, Samuel H. Amsterdam, Akshay A. Murthy, Hongfei Zeng, Tobin J. Marks, Vinayak P. Dravid, Mark C. Hersam, Nathaniel P. Stern

**Affiliations:** 1 Applied Physics Program, Northwestern University, Evanston, IL 60208 USA; 2 Department of Physics and Astronomy, Northwestern University, Evanston, IL 60208 USA; 3 Department of Chemistry and the Materials Research Center, Northwestern University, Evanston, IL 60208 USA; 4 Department of Materials Science and Engineering and the Materials Research Center, Northwestern University, Evanston, IL 60208 USA; 5 International Institute of Nanotechnology, Northwestern University, Evanston, IL 60208 USA; 6 NUANCE Center, Northwestern University, Evanston, IL 60208 USA; 7 Department of Electrical and Computer Engineering, Northwestern University, Evanston, IL 60208 USA

**Keywords:** Spintronics, Two-dimensional materials, Microresonators, Quantum information

## Abstract

Selective breaking of degenerate energy levels is a well-known tool for coherent manipulation of spin states. Though most simply achieved with magnetic fields, polarization-sensitive optical methods provide high-speed alternatives. Exploiting the optical selection rules of transition metal dichalcogenide monolayers, the optical Stark effect allows for ultrafast manipulation of valley-coherent excitons. Compared to excitons in these materials, microcavity exciton-polaritons offer a promising alternative for valley manipulation, with longer lifetimes, enhanced valley coherence, and operation across wider temperature ranges. Here, we show valley-selective control of polariton energies in WS_2_ using the optical Stark effect, extending coherent valley manipulation to the hybrid light-matter regime. Ultrafast pump-probe measurements reveal polariton spectra with strong polarization contrast originating from valley-selective energy shifts. This demonstration of valley degeneracy breaking at picosecond timescales establishes a method for coherent control of valley phenomena in exciton-polaritons.

## Abstract (web summary)

Microcavity exciton-polaritons in atomically thin semiconductors are a promising platform for valley manipulation. Here, the authors show valley-selective control of polariton energies in monolayer WS2 using the optical Stark effect, thereby extending coherent valley manipulation to a hybrid light-matter regime

## Introduction

Breaking spin degeneracy with light enables high-speed control of coherent states and forms the basis of emerging quantum technologies^1,2^. Degeneracy breaking enables coherent manipulation of spin by providing control over the relative phase in a quantum superposition. A classic example relies on Zeeman splitting of spin states in a magnetic field, but this approach is generally ill-suited for high-speed and spatially localized operations. Using circularly-polarized laser pulses, the optical Stark effect (OSE) can provide large effective magnetic fields for breaking spin degeneracy on sub-picosecond timescales, offering an all-optical alternative that enables more precise control of quantum states^1,2^.

The OSE is also a powerful tool for manipulating valley pseudospin in transition metal dichalcogenides (TMDs). In monolayer TMDs, a pseudospin arises from the K and K′ valleys in the band structure, which are degenerate in energy but separate in momentum space^3^. Because optical transitions in each valley are governed by polarization-sensitive selection rules, ultrafast optical Stark shifts can be used to break valley degeneracy^4,5^ and control valley pseudospin in excitons^6^, suggesting applications in quantum information. At the very least, polarization-sensitive Stark effects have continued to provide insights into valley exciton physics^7–9^, while opening broader possibilities for dynamic band engineering through valley-dependent control of energy levels.

Despite their potential, valley excitons suffer from limitations due to their small sizes^10^, short diffusion lengths^11,12^, and fast valley depolarization^13,14^. When TMDs are embedded in microcavities, strong light-matter coupling yields hybrid exciton-polaritons that preserve the valley pseudospin of the material, while also incorporating properties of light that can overcome many limitations of excitons. Exciton-polaritons exhibit reduced effective masses and faster propagation speeds favorable for long-distance information transfer^15^, as well as modified relaxation dynamics^16–20^ that enhance valley coherence^21,22^. Demonstrations of the optical valley Hall effect^15^, room-temperature valley coherence^22,23^ and valley-coherent manipulation^23^ in TMD polaritons further highlight how these hybrid quasiparticles enable valley phenomena that cannot be achieved with excitons alone. For many future applications, high-fidelity control of pseudospin in TMD polaritons requires ultrafast degeneracy-breaking of cavity-coupled valley states. Valley-selective optical Stark shifts in TMD polaritons could enable pseudospin state control in a regime that can access the desirable light-like properties of polaritons.

While the optical Stark effects of excitons have been measured in a variety of semiconductors, optical Stark effects in exciton-polaritons have so far only been measured in GaAs quantum wells^24–27^. Even in GaAs quantum wells, polarization-selective optical Stark shifts of polaritons have not yet been demonstrated. Expanding these observations to polaritons in different materials like TMDs is in itself an important step in verifying that polariton OSE physics can be broadly applied to describe the effect in polaritons with larger Rabi splitting, faster decay rates, and larger inhomogeneous broadening. Even more importantly, the different electronic and optical properties of TMDs can enable additional features of polariton optical Stark effects, like the previously unobserved polarization-selective polariton Stark shift.

**Fig. 1:**
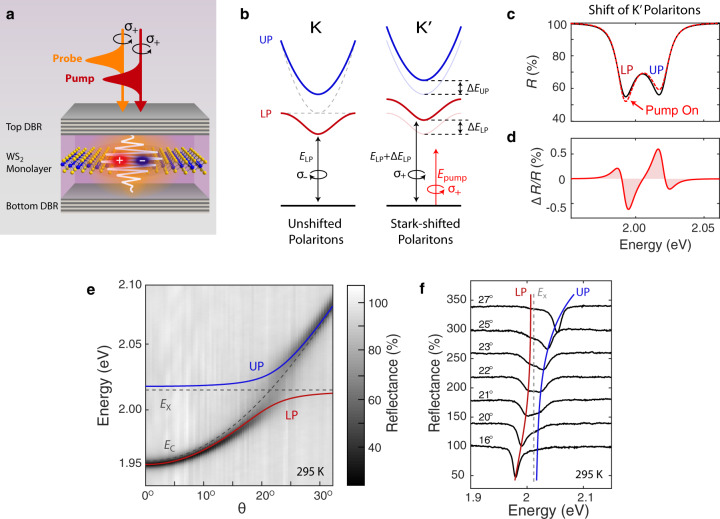
The valley-selective optical Stark effect in TMD microcavity exciton-polaritons. **a** Cartoon of the polarization-dependent transient reflectance measurement of WS_2_ polaritons in a monolithic cavity. **b** Schematic of valley-selective optical Stark shift for polariton energy dispersions. Polaritons at the K and K′ valleys couple to left-handed (*σ*_−_) and right-handed (*σ*_+_) circularly-polarized light, respectively. The *σ*_+_ polarized, below-bandgap pump only couples to polaritons in the K′ valley, inducing an optical Stark shift of both the upper (Δ*E*_UP_) and lower (Δ*E*_LP_) polariton energies in just this one valley. **c** Calculated change in microcavity reflectance *R* for polaritons in the K′ valley due to *σ*_+_-polarized pump pulse. The K valley polaritons should experience no change in reflectance. **d** Expected differential reflectance spectrum Δ*R*/*R* induced by the pump, showing the characteristic lineshape of the polaritonic optical Stark effect. **e** Momentum-space reflectance spectrum obtained by angle-resolved spectroscopy showing an avoided crossing when the cavity mode energy *E*_C_ is resonant with the TMD exciton energy *E*_X_. **f** Cross sectional reflectance data from **e** at various angles (offset for clarity). Both **e** and **f** show UP and LP branch dispersions in blue and red coinciding with the spectral peak energies.

In this report, we show how the optical selection rules of TMDs can enable optical Stark shifts of polaritons with valley-selectivity. Using ultrafast circularly-polarized transient reflectance, we demonstrate a valley-selective optical Stark effect in WS_2_ exciton-polaritons (Fig. 1a). A sub-picosecond blueshift of both upper and lower polariton branches when the pump and probe pulses are co-circularly polarized, with no appreciable shift when they are cross-circularly polarized, provides a signature of the effect. Valley-dependent energy shifts persist over a range of experimental conditions that enhance the light-like properties of polaritons, and the shifts are well-understood using a transfer matrix model of the coupled exciton-cavity structure. This robust, valley-selective control over the energy levels of TMD polaritons establishes a powerful approach for coherent manipulation of hybrid light-matter states with valley sensitivity.

## Results

### Optical Stark effect in WS_2_ exciton-polaritons

The polaritonic OSE results from an interaction between an exciton with energy *E*_X_ and two distinct optical fields: a near-resonant cavity photon mode with energy *E*_C_ ≈ *E*_X_, and sub-resonant light with energy *E*_P_ < *E*_X_. Strong coupling between the exciton and the cavity photon results in highly mixed polariton eigenstates that split into an upper and lower branch. Weaker coupling to the sub-resonant light results in an intensity-dependent Stark shift of both polariton branches^25^. Since TMD polaritons inherit the polarization-dependent selection rules from their excitonic component^16–18^, a circularly-polarized pump will induce a Stark shift of polaritons in only one valley, leaving polaritons in the opposite valley unperturbed (Fig. 1b). This valley-selective shift manifests as a change in the polariton reflectance spectrum: while the subresonant light is present, the upper polariton (UP) and lower polariton (LP) peaks shift to higher energy and change amplitude (Fig. 1c). Transient reflectance spectroscopy measures the difference between the shifted and unshifted reflectance spectra Δ*R*, normalized by the unshifted reflectance *R*. The expected Δ*R*/*R* signal shown in Fig. 1d has the “double Lorentzian-derivative” shape that characterizes the experimental signature of the polaritonic optical Stark effect^25,26^. For a valley-selective polaritonic OSE, this Δ*R*/*R* spectrum only occurs when the pump and probe are co-polarized.

To observe this phenomenon, planar microcavities supporting WS_2_ exciton-polaritons are fabricated using established procedures^28^ (see Supplementary Information). The monolithic microcavities consist of two SiO_2_/Si_3_N_4_ distributed Bragg reflectors, with a monolayer of WS_2_ embedded in the center. Using angle-resolved reflectance measurements of the momentum-space polariton dispersion, an avoided crossing is observed as the cavity energy (*E*_C_) is tuned through the exciton resonance (*E*_X_), indicating polariton formation (Fig. 1e, f). From the reflectance spectrum at zero cavity-exciton detuning (Δ = *E*_C_ − *E*_X_ = 0) we extract a typical Rabi splitting *ℏ*Ω_R_ = 26 meV between the LP and UP, comparable to previous reports of TMD exciton-polaritons^21,22,28,29^.

**Fig. 2:**
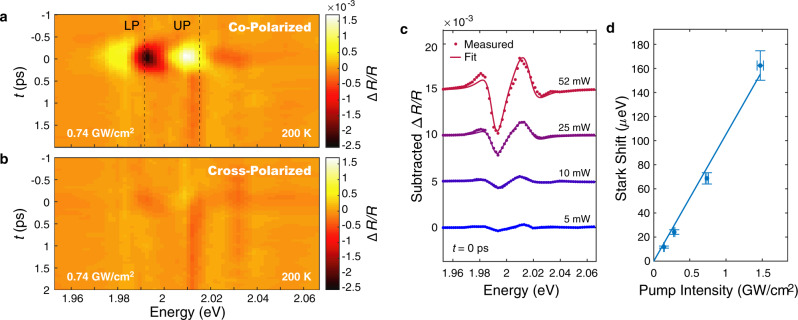
Pump-probe measurement of valley-selective optical Stark shift. **a** Pump-induced differential reflectance when pump and probe are co-circularly polarized. The sharp signal at *t* = 0 is attributed to a optical Stark shift of both upper and lower polaritons. **b** Differential reflectance when pump and probe are cross-polarized, lacking sharp signal at *t* = 0. **c** Background-subtracted Δ*R*/*R* signal at zero delay for increasing pump intensities (dots) and fits (solid) to a transfer matrix model. **d** Stark shift magnitude extracted from fits to data in **c** showing expected linear trend. Error bars in pump intensity represent uncertainty in the measured pump power and spot size. Error bars in Stark shift magnitude represent uncertainty from the fit procedure (see Supplementary Information).

The valley-selective exciton-polariton OSE is demonstrated using polarization-resolved pump-probe spectroscopy. A sub-picosecond, 1.493 eV pump pulse induces a change Δ*R*/*R* in the microcavity reflectance spectrum measured by a low-intensity energy-tunable probe pulse. Both pump and probe are circularly-polarized and normally incident on the sample. Figure 2a, b shows Δ*R*/*R* as a function of pump-probe delay time *t*. Since *E*_X_ is temperature-dependent, the detuning Δ can be minimized near *T* = 200 K. At this temperature, UP and LP reflectance features have roughly equal spectral weight with Rabi splitting *ℏ*Ω_R_ = 24 meV (Supplementary Fig. 8). When the pulses are co-circularly polarized, a sharp signal is observed at *t* = 0 for the duration of the pump-probe temporal overlap. The spectral response is characteristic of the anticipated polaritonic optical Stark shift (Fig. 1c, d), and the pulsewidth-limited signal indicates that the shift only occurs when the pump pulse is present. The lack of coherent oscillations before *t* = 0 confirms that the measurement is in the adiabatic limit of the Stark shift, which avoids additional Δ*R*/*R* spectral broadening^26^. When the pump and probe are cross-circularly polarized there is no significant signal near *t* = 0. This dramatic polarization contrast suggests that for a particular circular polarization of the off-resonant pump, the Stark shift is isolated to polaritons in a single valley.

The long-timescale background signal present for *t* > 0 in both polarization configurations is attributed to the generation of real carriers by two-photon absorption of the pump pulse^4,26,30^. Since the OSE signal here is polarization-selective, the background is removed for subsequent analysis by subtracting the cross-polarized signal from the co-polarized signal. This subtracted Δ*R*/*R* spectrum is used as a proxy for the underlying circular dichroism caused by the valley-selective shift.

We model the Stark shift of the polaritons by only considering the energy shift of the neutral exciton Δ*E* relative to the fixed cavity energy. For the small energy shifts measured in this report, this perturbative approach is an accurate description of the polaritonic OSE^25^. An in-depth discussion of the perturbative regime in relation to the full Jaynes–Cummings Hamiltonian is provided in the Supplementary Information. However, most of the quantities reported in this manuscript do not explicitly rely on the Jaynes–Cummings Hamiltonian; our analysis only considers pump-induced changes to the exciton energy, and models the associated change in cavity reflectance. The Jaynes–Cummings Hamiltonian is only used to interpret the measured spectral parameters as UP and LP shifts vs. temperature. Additionally, for the sample temperatures above 120 K measured in this report, the impact of the trion is expected to be small^28^, and is not included in our simple model. The potential influence of the trion on the polariton spectra at lower temperatures is also discussed in the Supplementary Information.

The magnitude of the Stark shift Δ*E* is proportional to the ratio of the peak pump intensity *I*_P_ and the pump detuning Δ_P_ = *E*_P_ − *E*_X_ as ${{\Delta }}E\propto \frac{{I}_{{{{\rm{P}}}}}}{{{{\Delta }}}_{{{{\rm{P}}}}}}$^30^. We confirm the expected linear dependence on *I*_P_ by repeating the experiment at pump intensities spanning an order of magnitude (Fig. 2c). Δ*E* is extracted by fitting the pump-induced Δ*R*/*R* spectra at *t* = 0 to a transfer-matrix model of the exciton-cavity system. In this model, the dielectric function of WS_2_ is represented by a single oscillator at the A exciton that is allowed to shift in energy (see “Methods” section for additional details). The Stark shifts extracted from the fits are shown as a function of pump intensity in Fig. 2d. Δ*E* shows a clear linear trend, with a maximum shift of 161 μ eV for a 1.47 GW/cm^2^ pump. We emphasize that the linear trend is seen for the exciton shift parameter Δ*E* extracted from a fit to the Δ*R*/*R* spectrum, which goes beyond the simple observation of a change in spectral amplitude due to increased laser power. As shown in Supplementary Fig. 7, the μeV-scale Stark shift causes only a small change in the photon and exciton fractions of the polariton (given by the Hopfield coefficients). Still, we can clearly measure and fit the spectral signature of the polaritonic Stark shift, highlighting that even small changes in polariton composition lead to spectral changes that can be detected using our approach of pump-probe spectroscopy and reflectance modeling of the cavity structure.

### Valley-selective shifts of detuned polaritons

The key advantage of polaritons over excitons is that they share the valley selectivity of excitons and the coupling to traveling photons provided by their half-light nature. The relative mixture of photon and exciton in a polariton depends on the exciton-photon detuning Δ, allowing tailored polariton characteristics for different applications. The Hopfield coefficients are calculated as a function of detuning in Supplementary Fig. 7. At large detuning, one of the polariton branches can exhibit markedly more photon-like properties, such as drastically reduced linewidths, longer coherence lengths, and faster relaxation rates that have advantages for long-distance information transfer^31^. However, the reduced exciton proportion of such off-resonant polaritons could restrict the ability for optical manipulation through the Stark effect.

**Fig. 3:**
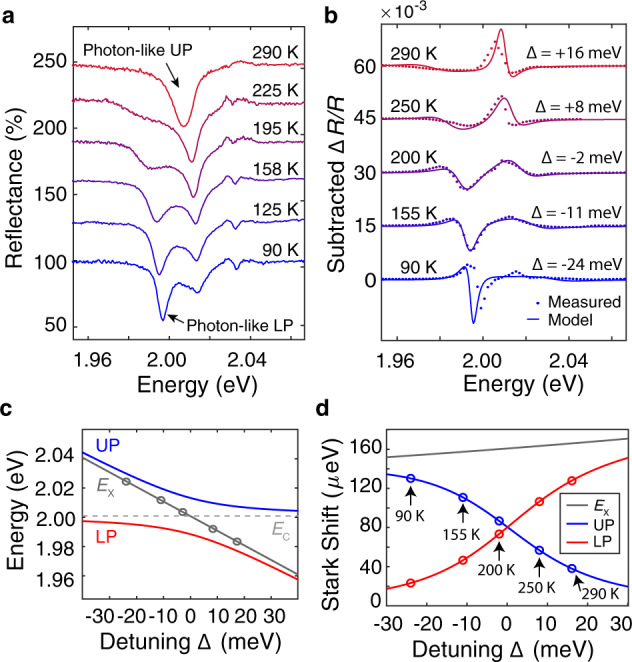
Valley-sensitive Stark shift over wide range of detunings. **a** Polariton doublet as a function of temperature offset by 30% intervals. Varying temperature tunes polaritons from positive to negative detuning regime, where UP is more photon-like at high temperatures and LP is more photon-like at low-temperatures. The spectra are normalized to reflectivity of the bare cavity, resulting in a small artifact near the 2.037 eV resonant energy of the bare cavity. **b** Δ*R*/*R* spectra at various Δ induced by 1.47 GW/cm^2^ pump, offset by 0.015 intervals. The spectra are well-described by the Stark shift model, indicating that the valley-selective polariton OSE persists across detunings. **c** Model of polariton energies as exciton tunes with temperature. Valley-selective Stark shift measured at cavity-exciton detunings Δ indicated by gray circles. **d** Stark shift of the upper and lower polariton branches inferred from analysis using Jaynes–Cummings Hamiltonian. The magnitude of the Stark shift tracks the excitonic Hopfield coefficient.

Because the exciton energy of the TMD shifts with temperature but the microcavity photon resonance is nearly constant, it is possible to explore the valley-sensitive OSE in different polariton detuning regimes in the same sample. Changing detuning with sample temperature, rather than by changing the probe angle, allows us to hold the spot size and location constant, which minimizes the impact of sample inhomogeneity (see Supplementary Fig. 8). Temperature tuning provides continuous variation of detuning Δ from a photon-like UP at room temperature to a photon-like LP at *T* = 90 K (Fig. 3a). We repeat the polarization-dependent transient reflectance measurement over this range of sample temperatures to investigate retention of OSE valley selectivity across polariton detunings (Fig. 3b). For all temperatures, we observe a pump-induced signal with strong circular-polarization contrast, showing that the valley OSE enables polarization-selective control of polaritons even in regimes of moderate detuning. The Δ*R*/*R* spectra are again well-described by the polariton model that accounts for temperature-dependent *E*_X_ (Fig. 3c). Allowing for variation in the exciton oscillator parameters caused by their temperature dependence further improves the fit to the data (Supplementary Fig. 8). The excellent agreement between the modeled and measured spectra clearly establishes that the valley Stark shift is the dominant effect governing the observed features across temperatures.

We estimate the shift of each individual polariton branch by inputting the measured Rabi splitting, induced exciton Stark shift magnitude, and cavity-exciton detuning into a modified Jaynes–Cummings Hamiltonian (see Supplementary Information). The calculated Stark shifts for the UP and LP at different temperatures are shown in Fig. 3d, showing how the magnitude of the shift of a given polariton branch increases with increasing exciton character. This feature is not immediately obvious from the differential reflectance spectra because the polariton linewidths are wide and the energy shifts are small. Additional discussion of the relationship between the spectral features and the Jaynes–Cummings analysis can be found in the Supplementary Information.

**Fig. 4:**
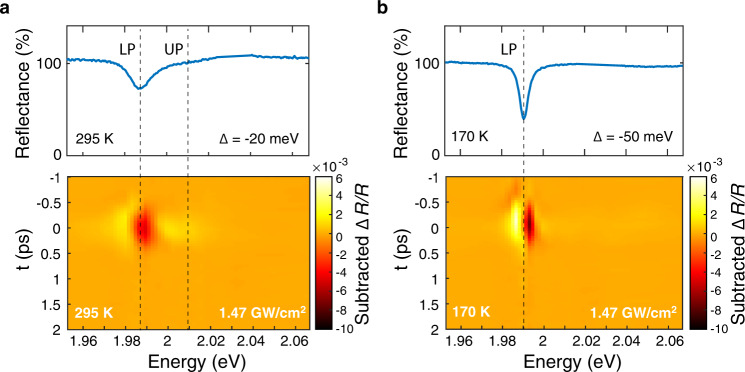
Valley-selective shift of highly-detuned polaritons. **a** Reflectance spectrum (top) of a photon-like lower polariton at room temperature. The small UP feature is barely discernible near 2.01 eV. Pump-induced shift of the LP dominates the polarization-dependent response of the Δ*R*/*R* spectrum (bottom). **b** Same sample at 170 K, where the exciton is further detuned from the cavity. The LP reflectance feature is narrower and deeper due to its more photon-like character and UP is no longer visible. The pump-induced Δ*R*/*R* spectrum continues to exhibit strong polarization contrast in this regime, demonstrating how the optical Stark shift of a highly photonic WS_2_ polariton still maintains valley selectivity due to its small excitonic character.

We next extend the OSE detuning dependence to a limit in which only a single photon-like polariton is observable. We use a different sample, where the cavity photon resonance is designed to have a larger detuning from the exciton energy. The observable polariton in this highly-detuned regime is similar to the bare cavity mode, but it maintains a small excitonic fraction typical of a weakly coupled light-matter dressed state. Although this excitonic component is small, it can provide a means to manipulate the cavity response using the unique features of the TMD material. Rapid modulation of a single cavity resonance has important applications in telecommunications and information processing^32^. The valley selection rules of TMD polaritons provide an additional polarization degree of freedom for such cavity modulation, with relevance for spin-based photonics^33^. As a proof of principle, we demonstrate how the optical Stark effect remains valley-selective for TMD polaritons even in this highly-detuned limit. A detailed analysis of detuning and estimations of the Stark shift magnitude in these measurements are provided in Supplementary Fig. 9. Figure 4a shows the response of a detuned polariton sample at room temperature, where the UP feature is barely visible in reflectance and the Stark shift signal is dominated by the LP. Here the excitonic fraction is ~17% (see Supplementary Fig. 7 for Hopfield analysis). Lowering the sample temperature to 170 K increases the detuning so only a single, highly-photonic LP resonance is visible, with an excitonic fraction of ~5% (Fig. 4b). The pump-induced Δ*R*/*R* signal is isolated to the LP and maintains high polarization contrast. This highlights how the small excitonic fraction of the LP still imparts useful optical selection rules on highly-photonic polariton states, and demonstrates how the valley polariton Stark effect can be used to induce polarization-selective shifts in a single cavity resonance on sub-picosecond timescales.

## Discussion

Combining ultrafast optics with 2D material polaritonics, our work establishes an approach for state manipulation in hybrid light-matter systems by demonstrating how the optical Stark effect can break valley degeneracy in exciton-polaritons. The effect remains valley-selective over a wide range of cavity-exciton detunings, paving the way towards coherent control of valley in regimes that accentuate the light-like properties of polaritons. In bare TMD excitons, circularly-polarized pump fields tuned above^34^, below^8,35^, and on-resonance^9^ with various exciton transitions have revealed valley-dependent couplings between optical fields and different exciton species. Similarly, the valley-sensitivity of the polaritonic Stark shift established in this work should extend to alternative pumping regimes, creating rich opportunities to explore additional valley-dependent couplings in 2D polaritons.

In this report, we are limited to a perturbative regime of the Stark shift due to our highly-detuned pump. By tuning the pump closer to the polariton energies, significantly larger Stark shifts can be expected in which the magnitude of the shift exceeds the Rabi splitting, and the system can no longer be described perturbatively^27^. When pumping near-resonance, many-body interactions between virtual excitons created by the driving field can also begin to dominate the observed Stark shifts, manifesting as an anomalous blue-shift when the pump is tuned slightly above resonance^34^. In polaritons with larger spatial size, the more significant overlap of polariton wavefunctions could lead to stronger repulsive Coulombic interactions between particles. This could cause many-body interactions to become dominant over a wider range of detunings, potentially yielding even larger anomalous blue-shifts when pumping above resonance. Additionally, there have been exciting theoretical predictions for spectral signatures of quantum many-body correlations in microcavity polaritons, when the driving field is resonant with the lower polariton branch^36^. Future experiments eliminating the pump energy constraint could determine the role of many-body interactions between valley polaritons in these currently unmeasured near-resonance pumping regimes. In this way, the valley-selective optical Stark effect in exciton-polaritons lays the groundwork for a broad exploration of coherent valley phenomena in 2D material polaritons and their technological implications.

## Methods

### Angle-resolved reflectance

Angle-resolved reflectance measurements are used to characterize the momentum-space dispersion of the exciton-polaritons. These measurements are performed by imaging the back focal plane (Fourier plane) of a 50 × 0.6 NA microscope objective onto the entrance slit of a spectrometer that is equipped with a CCD. This allows us to measure both the spectral (CCD *x*-axis) and angular (CCD *y*-axis) dependence of the cavity sample simultaneously in a single measurement. Our approach follows that of ref. ^28^.

### Time-resolved measurement

For the transient reflectance measurements, pump pulses are produced by a Ti-sapphire laser (Coherent MIRA-900F) that emits sub-ps pulses at 1.493 eV with a repetition rate of 76 MHz. Part of this beam is picked off to drive a passive optical parametric oscillator (MIRA-OPO), which generates probe pulses that are tuned over the polariton energies from 1.95 to 2.06 eV. This probe source uses a nonlinear optical crystal that is optimized for the 1.493 eV energy of the synchronous pump pulses. As such, the pump laser must be fixed at 1.493 eV for all measurements. The circular polarization of the pump and probe are controlled independently to establish the polarization-dependence of the Stark shift response.

For all measurements, the 1.493 eV pump is detuned from the polariton resonance by ~520 meV. This significant detuning puts the pump outside of the stop band of the cavity, enabling pumping at normal incidence without losing a significant amount of pump power due to reflection off of the top DBR. The pump and probe beams are co-linear and are focused on the sample with an f50 achromatic doublet lens to a spot size of 13 μm and 10 μm, respectively. The pump has a peak intensity of 1.47 GW/cm^2^ and the probe has a much lower peak intensity of 0.3 MW/cm^2^. The bandwith of the probe pulse is ~9 meV. In order to better resolve the spectral features of the Stark shift signal, we use a monochromator (Edmund Mini-Chrom) with 100 μm slits to spectrally filter the reflected probe pulses down to a bandwidth of ~3 meV before collecting the signal on an avalanche photodiode. The pump is chopped at 100 kHz using a photoelastic modulator while the probe is modulated at 1033 Hz with a mechanical chopper. We measure Δ*R*/*R* using two lock-in amplifiers to extract the pump-induced signal Δ*R* at 100 kHz and the reflectance of the probe *R* at 1033 Hz. Additional details on the measurement can be found in Supplementary Fig. 3.

### Model of Stark shift

To fit the Δ*R*/*R* spectra induced by the optical Stark effect, the dielectric function of WS_2_ is represented by a single oscillator at the A exciton. We insert this dielectric function into a transfer-matrix model of the cavity reflectance. With other oscillator parameters determined beforehand, the only free parameter allowed is the energy shift of the A exciton resonance. This simple, one-parameter model effectively captures the salient features of the polaritonic Stark shift and provides excellent agreement with the measured Δ*R*/*R* data.

For the temperature-dependent Δ*R*/*R* spectra, we model the exciton oscillator using the best-fit parameters found from the fits at 200 K, and model the shift of the exciton center energy at different temperatures using the slope of a linear fit to the temperature-dependent PL of WS_2_. Since the pump energy is constant in our experiments, the pump-exciton detuning Δ_P_ also changes slightly (<10%) with temperature. To account for the change in Δ_P_, the magnitude of the Stark shift used to calculate the Δ*R*/*R* spectra is changed from Δ*E* = 154 μeV at 90 K to Δ*E* = 167 μeV at 290 K in accordance with the 1/Δ_P_ scaling of the Stark shift. Additional details on the model, fitting procedures, and a discussion of error are provided in Supplementary Information.

## Supplementary information

Supplementary Information

## Data Availability

All data relevant to this work are available on request to the corresponding author.
