# Supplementary material for: Valley-selective optical Stark effect of exciton-polaritons in a monolayer semiconductor
Source: Nat Commun. 2021 Jul 26;12:4530. doi: 10.1038/s41467-021-24764-8 (PMC8313563; doi:10.1038/s41467-021-24764-8)
Supplement: Supplementary file 1 — Supplementary Information [file 41467_2021_24764_MOESM1_ESM.pdf]

Valley-selective optical Stark effect of exciton-polaritons in a monolayer semiconductor

Trevor LaMountain,[1] Jovan Nelson,[1] Erik J. Lenferink,[2] Samuel H. Amsterdam,[3] Akshay A. Murthy,[4,5] Hongfei Zeng,[2] Tobin J. Marks,[3,4] Vinayak P. Dravid,[4,5,6] Mark C. Hersam,[1,3,4,7] and Nathaniel P. Stern[1,2,*]

[1]*Applied Physics Program, Northwestern University, Evanston, Illinois 60208, USA*

[2]*Department of Physics and Astronomy, Northwestern University, Evanston, Illinois 60208, USA*

[3]*Department of Chemistry, and the Materials Research Center, Northwestern University, Evanston, Illinois 60208, USA*

[4]*Department of Materials Science and Engineering, and the Materials Research Center, Northwestern University, Evanston, Illinois 60208, USA*

[5]*International Institute of Nanotechnology, Northwestern University, Evanston, Illinois 60208, USA*

[6]*NUANCE Center, Northwestern University, Evanston, Illinois 60208, USA*

[7]*Department of Electrical and Computer Engineering, Northwestern University, Evanston, Illinois 60208, USA*

(Dated: June 17, 2021)

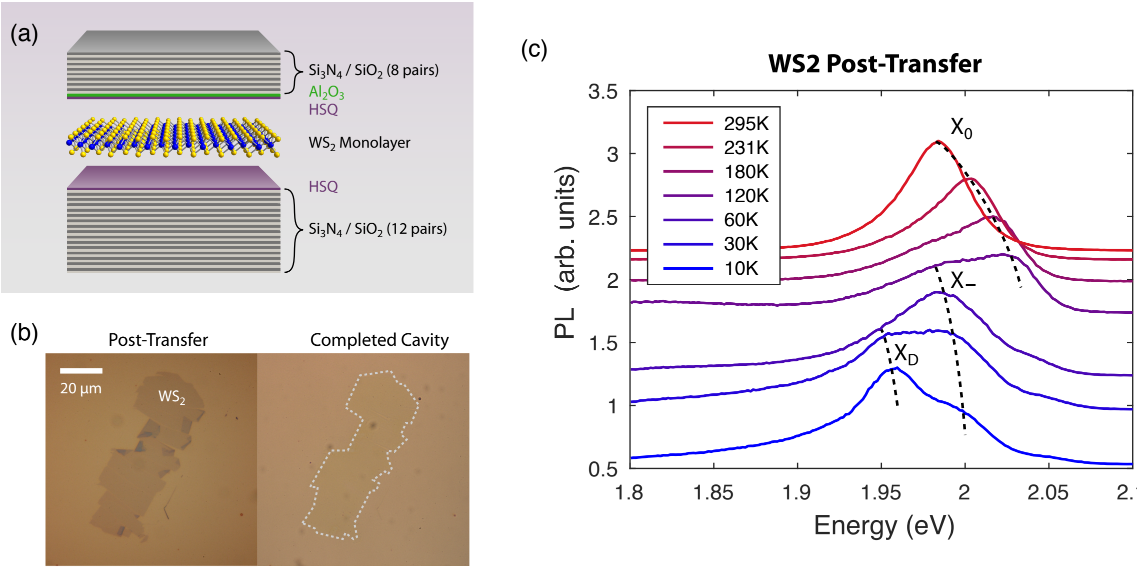

Supplementary Fig. 1. (a) Detailed schematic of cavity sample. (b) Optical images of region of cavity containing tape-exfoliated WS_2 monolayer before and after deposition of top Al_2O_3, HSQ, and DBR structure. (c) Temperature dependence of normalized tape-exfoliated WS_2 photoluminescence after transfer onto the bottom DBR structure. At higher temperatures the neutral exciton (X_0) dominates, whereas below ∼100 K trion emission (X_-) and defect-localized exciton emission (X_D) dominate.

SAMPLE FABRICATION AND CHARACTERIZATION

The distributed Bragg reflectors (DBRs) used to form the optical cavity consist of alternating layers of Si_3N_4/SiO_2 and were grown using plasma-enhanced chemical vapor deposition [Supplementary Fig. 1(a)]. The top and bottom DBRs cosist of 8.5 and 12.5 pairs, respectively. The center region consisting of SiO_2 is designed to have an optical path length of approximately half the designed cavity wavelength. Once the bottom DBR was grown, a layer of hydrogen silsesquioxane (HSQ) was spin-coated and annealed to form the bottom half of the $\lambda/2$ cavity [1]. The WS_2 monolayer was then transferred onto the bottom half of the cavity structure using a polymer-based transfer method similar to that used in Ref. [2] [Supplementary Fig. 1(b)]. The optical characteristics of the monolayer were then characterized via temperature-dependent photoluminescence (PL) [Supplementary Firgure 1(c)]. In our samples, the neutral exciton (X_0) dominates above ∼120 K, while other charged and defect-bound excitons become significant at lower temperatures. Following PL characterization,

the top half of the cavity structure was deposited. This began with another spin-coated layer of HSQ to form the top half of the $\lambda/2$ cavity, and was followed by a $\sim$7 nm layer of Al_2O_3 is deposited by atomic layer deposition to protect the monolayer from plasma damage during subsequent CVD growth [1]. Al_2O_3 was deposited by atomic layer deposition in an Anric AT 400 reactor using cycles of trimethylaluminum and water at 150 C. The deposition rate was calibrated to 1.2 Å/cycle using ellipsometry. The top 8-pair DBR was then grown by CVD.

Multiple samples were fabricated using this approach, some using tape-exfoliated monolayers sourced from bulk crystal (HQ Graphene) and others using WS_2 monolayers grown by CVD. Each sample was designed with a slightly different cavity resonance near the neutral exciton energy E_X, and all samples exhibited anticrossing [Supplementary Fig. 2].

The monolayer flakes of WS_2 grown by CVD used a metal oxide-based CVD growth method. This reaction was conducted within a 1" diameter quartz tube that was placed inside a three-zone tube furnace (MTI OTF-1200X). 10 mg of WO_3 powder (Sigma Aldrich) was spread evenly about a Si wafer capped with a 300 nm SiO_2 layer and positioned face-up at the center of the quartz tube. A second identical SiO_2/Si wafer is suspended face-down approximately 1-2 mm above the first. 500 mg of sulfur powder was additionally placed in an alumina boat at an upstream location 22 cm from the hot center. The center of the furnace was then heated to 830 C. Through heat diffusion from the hot center, the temperature at the alumina boat containing sulfur powder reaches 200 C. Throughout the reaction, 100 sccm ultra high purity Ar gas is flowed through the quartz tube. The following temperature profile was used: 50 minute ramp to 550 C, followed by a 30 minute ramp to 830 C, followed by a natural cool-down.

ADIABATIC REGIME OF TIME-RESOLVED MEASUREMENT

When the pump pulse duration t_{P} is significantly shorter than the Rabi cycle period $t_{\mathrm{R}} = 4\pi/\Omega$ of the polariton, the signature optical Stark spectrum can broaden and become convoluted with other coherent oscillatory signals [3, 4]. Ensuring that $t_{\mathrm{P}} > t_{\mathrm{R}}/2$ avoids these complicating features [4]. Our measurements satisfy this condition, where $t_{\mathrm{P}} \sim 375$ fs and the 24 meV Rabi splitting of our sample corresponds to $t_{\mathrm{R}} = 345$ fs. Moreover, the measured $\Delta R/R$ signal exhibits a narrow bandwidth and a lack of coherent spectral or

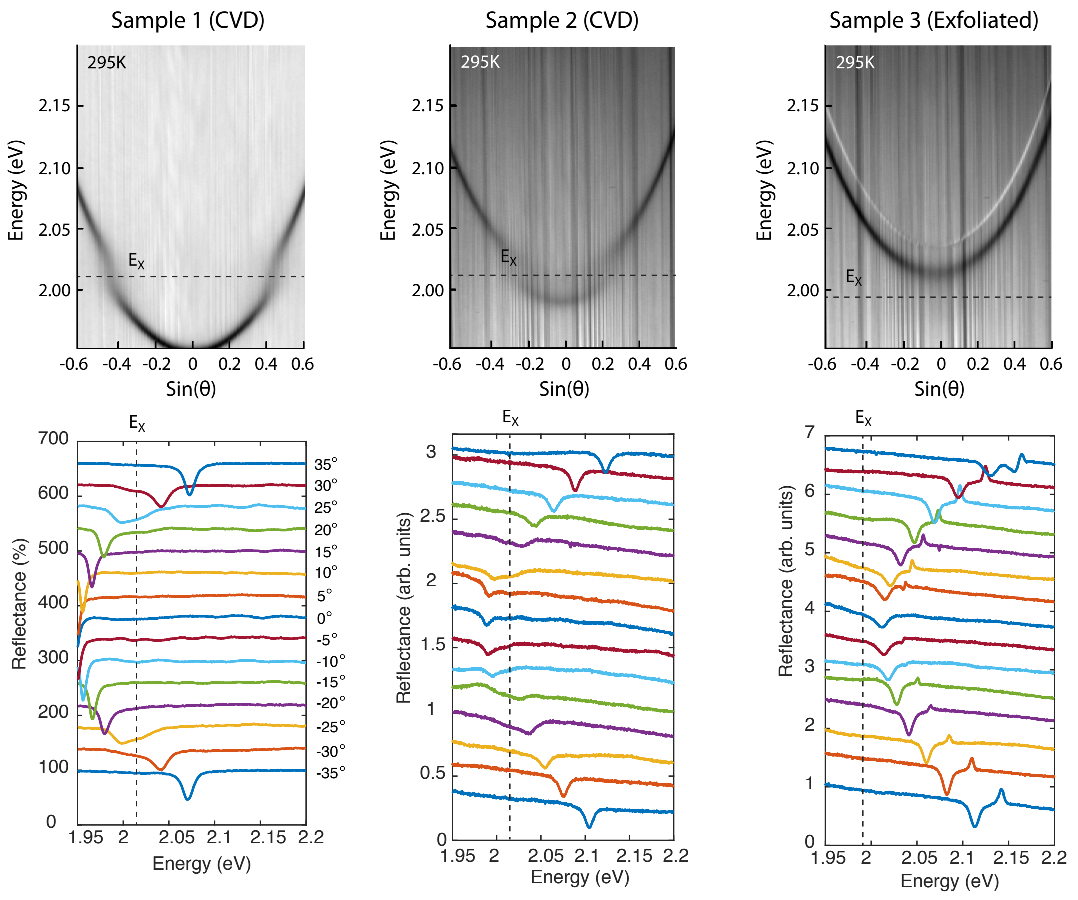

Supplementary Fig. 2. (top row) Fourier reflectance spectra for multiple samples showing anti-crossing near exciton resonance E_X. Sample 1 is shown in Fig. 1(e) of the main body, Sample 2 shown in Fig. 4 of the main body, and Sample 3 is shown in Fig. 2 and Fig. 3 of the main body. The exciton energy of the tape-exfoliated sample is lower than that of the CVD WS_2 samples. Reflectance not normalized for Samples 2 and 3. Bright curve in Sample 3 is an artifact from the spot sampling a region without WS_2. (bottom row) Cross-sections of the fourier reflectance spectra at different angles.

temporal oscillations before zero-delay.

DOMINANCE OF PHOTON-LIKE BRANCH IN $\Delta R/R$ SIGNAL

In the minimally-detuned regime at 200 K, the differential $\Delta R/R$ spectrum of Fig. 3 in the main paper shows two clear features corresponding to the UP and LP. At moderate de-

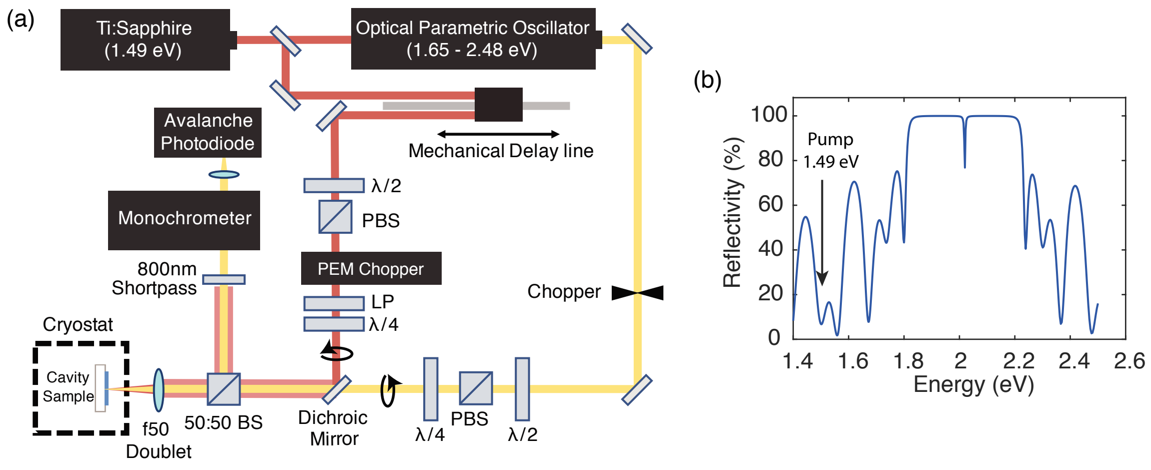

Supplementary Fig. 3. Details of transient reflectance measurement. (a) Schematic of the pump-probe measurement. (b) Simulated reflectance spectrum of 8p/12p cavity at normal incidence, showing that the highly-detuned pump is outside of the cavity stopband.

tunings, the $\Delta R/R$ spectrum is dominated by the response of the more photon-like polariton branch. This is not because the shift of the photonic branch is larger. Instead, this larger, sharper signal is a consequence of the deeper reflectance feature of the photon-like branch. In essence, the ΔR numerator of the photon-like branch gets smaller, but the reduced R value in the denominator leads to an amplified total signal. For this reason, the magnitude of the $\Delta R/R$ signal is not directly indicative of the size of the shift in this regime.

PERTURBATIVE MODEL OF POLARITONIC STARK SHIFT

The polaritonic optical Stark shift can be understood using the full Hamiltonian of the system, which accounts for the coherent interaction of the exciton with the cavity photon as well as the pump photon, or using a perturbative approach, where the OSE is modeled as a shift in only the excitonic component of the polariton. Ref. [5] has shown that for small Stark shifts, the eigenenergies of the full Hamiltonian differ only slightly from those in the perturbative approach. For the < 1 meV shifts measured in this work, the experimental error exceeds the the difference between these two approaches ($< 3\%$). As such, we can accurately model the polaritonic Stark shift as simply a shift in the exciton energy. To relate this perturbative model to the measured spectra, we use a transfer-matrix method to calculate the pump-induced change in reflectance in our microcavity structure. Specifically,

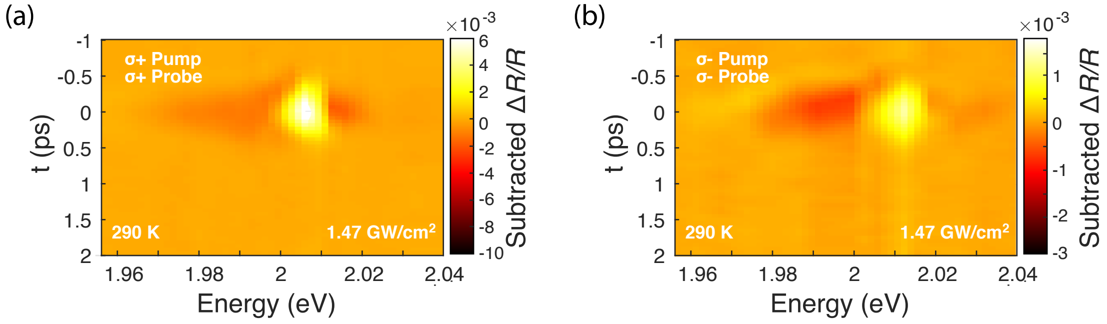

Supplementary Fig. 4. Consistency of signal for opposite pump helicity. (a) Pump-induced $\Delta R/R$ signal for right-circularly-polarized (σ_+) pump and probe. This is the full time-trace of the 290 K data presented in Fig. 3c of the main manuscript. (b) Pump-induced $\Delta R/R$ signal for left-circularly-polarized (σ_-) pump and probe, showing consistent spectral features as panel (a). The two measurements were taken at slightly different sample locations. For the measurement in panel (b), there was a slight misalignment of the pump spot relative to the probe spot, leading to a reduced effective fluence and hence a reduced magnitude of the signal.

we model the dielectric function of the WS_2 as a single Lorentz oscillator at the A exciton resonance, and allow the oscillator only to shift in energy. This simple phenomenological model effectively captures the salient features of the $\Delta R/R$ spectra induced by the Stark shift with excellent agreement between the model and the measurement. Fitting the measured spectra to this model, we extract a maximal 161 µeV shift for the exciton oscillator using a pump fluence of 1.47 GW/cm^2, corresponding to a 70 µeV UP shift and a 90 µeV LP shift.

The Hamiltonian that describes the polaritonic optical Stark effect accounts for the coupling between the WS_2 exciton and the cavity photon as well as the additional pump photon. The three-mode Jaynes-Cummings-type Hamiltonian for this system is:

$$H = \begin{pmatrix} E_{\mathrm{X}} & \hbar g & \hbar g_{\mathrm{P}} \\ \hbar g & E_{\mathrm{C}} & 0 \\ \hbar g_{\mathrm{P}} & 0 & E_{\mathrm{P}} \end{pmatrix} \tag{1}$$

where E_{X}, E_{C}, and E_{P} are the energies of the uncoupled exciton, cavity photon, and pump photon, respectively. $\hbar g = d|\mathcal{E}_{\mathrm{C}}|$ characterizes the coupling strength of the cavity photon, where d is the dipole matrix element of the neutral exciton and $\mathcal{E}_{\mathrm{C}}$ is the cavity field amplitude. Similarly $\hbar g_{\mathrm{P}} = d|\mathcal{E}_{\mathrm{P}}|$ characterizes the coupling strength of the pump photon [5]. When the pump field is zero, this reduces to the 2×2 Hamiltonian that describes polariton

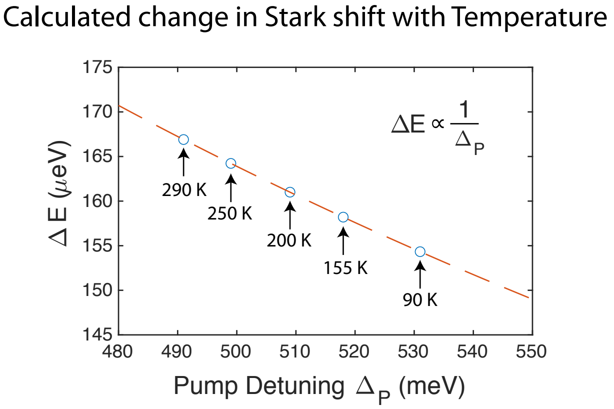

Supplementary Fig. 5. Calculated change in Stark shift magnitude at different sample temperatures used to calculate the expected $\Delta R/R$ spectra in Fig. 3c of the main manuscript. Since the exciton shifts with temperature, but the pump remains fixed at 1.493 eV, this causes a slight change in the pump-exciton detuning over the measured temperature range. Because the magnitude of the Stark shift scales with pump detuning as $\Delta E \propto 1/\Delta_{\mathrm{P}}$, there is a corresponding change in the magnitude of the shift. For our highly-detuned pump, the calculated change is minimal, with ΔE varying by $< 10\%$ over the measured temperature range.

formation. The polaritonic eigenenergies of this "pump off" Hamiltonian are shown as a function of detuning $\Delta = E_{\mathrm{C}} - E_{\mathrm{X}}$ in Supplementary Fig. 6(a). For nonzero pump fields, the interaction with the pump photon results in small shifts in the polariton eigenenergies.

If the pump field amplitude is significantly less than the cavity photon field, the induced shifts remain small with respect to the Rabi splitting $\hbar\Omega = 2\hbar g$. In this regime, we can calculate the polariton eigenenergies perturbatively by only considering the Stark shift of the exciton energy for a given pump field amplitude. The Stark-shifted exciton energy $E_{\mathrm{X}} + \Delta E$ is then inserted into the cavity-exciton Hamiltonian that omits the pump field interaction:

$$H_{cav} = \begin{pmatrix} E_{\mathrm{X}} + \Delta E & \hbar g \\ \hbar g & E_{\mathrm{C}} \end{pmatrix} \tag{2}$$

which results in shifted polariton eigenenergies. The relationship between exciton Stark shift ΔE and the shift of the UP and LP at zero detuning are shown in Supplementary Fig. 6(b), calculated using the full Hamiltonian and the perturbative approach. The associated error is plotted in Supplementary Fig. 6(c), showing how for pump-induced exciton shifts < 2 meV the discrepancy between the full and perturbative approaches is $< 3\%$.

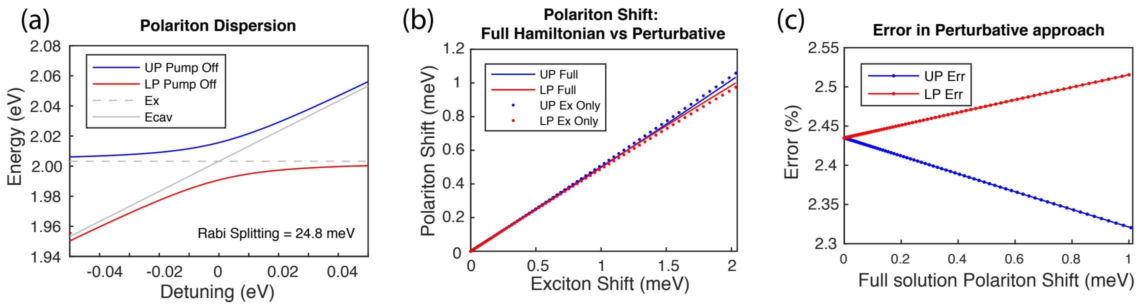

Supplementary Fig. 6. Comparison of modeling approaches. (a) Dispersion of polariton branches when pump is off. (b) Comparison of the size of the Stark shift of exciton-polaritons for a cavity at zero detuning. Solid lines are polariton shifts calculated from the full Hamiltonian, dotted lines are calculated using the perturbative approach. (c) Error in calculated UP and LP energies using the perturbative approach for increasing pump fields (represented by magnitude of uncoupled exciton shift). Error increases with larger pump fields (larger exciton shifts).

CHANGE IN HOPFIELD COEFFICIENTS DUE TO STARK SHIFT

To better understand the physics governing the polaritonic Stark shift, it is insightful to consider the details of how the OSE modifies the polariton eigenstates in the Jaynes-Cummings model (Eq. 1). The interaction with the pump field not only induces shifts of the polariton eigenenergies, but also changes the exciton and photon fractions (Hopfield Coefficients) of the polaritons. The effect of detuning on the polariton shifts [Supplementary Fig. 7(a)] is relatively straightforward: since the excitonic component of the polariton mediates the OSE, the magnitude of the induced shift of each polariton branch scales with the excitonic fraction, which changes as a function of detuning [6] [Supplementary Fig. 7(b)]. The pump-induced change in the Hopfield coefficients [Supplementary Fig. 7(c)] is less obvious, but can be intuitively understood using the perturbative framework. In the perturbative model, the coupling between the pump photon and the exciton induces a shift of the exciton energy relative to the cavity photon. Since the Hopfield coefficients change with detuning, this small pump-induced detuning leads to a change in the composition of the polariton, which is maximized at $\Delta = 0$ where the slope is maximal.

It is worth noting that for the "doubly photon dressed" solutions to the full Hamiltonian, there is some mixing between the exciton and the highly-detuned pump photon. Consequently, there is an additional coefficient associated with the pump photon fraction of the

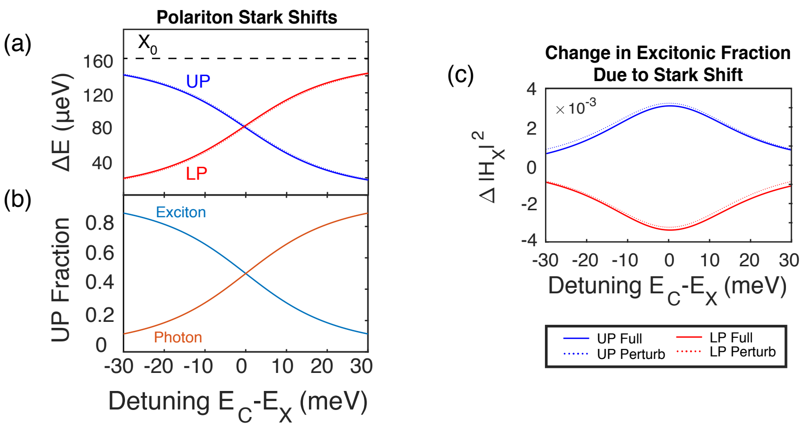

Supplementary Fig. 7. Analysis using Jaynes-Cummings Hamiltonian. (a) Stark shift of upper and lower polaritons corresponding to a 160 µeV shift of the neutral exciton. Dashed line are values calculated from perturbative approach (Eq. 2), solid lines from the full Jaynes-Cummings Hamiltonian (Eq. 1). (b) Relative photon and exciton fraction (Hopfield coefficients) of the upper polariton as a function of detuning calculated from Eq. 2 when $\hbar\Omega_R = 24$ meV. (c) Change in excitonic Hopfield coefficients of UP and LP induced by Stark shift. Maximal change at zero detuning.

mixed eigenstate. For the highly detuned (~ 520 meV) pump used in this work, the pump photon fraction of the polariton eigenstates is $< 1\%$, so it is ignored in our analysis. However, this mixing would be significant for a near resonant pump energy, and should be considered carefully in any future experiments.

FIT TO LORENTZ OSCILLATOR MODEL

We fit the measured $\Delta R/R$ spectrum with a model based on the transfer-matrix method that uses the perturbative approach to account for the polaritonic Stark shift. The dielectric function of the WS_2 monolayer in the cavity is modeled as a single Lorentz oscillator at the neutral exciton energy. Because there is inhomogeneity across the monolayer on the scale of the probe size, the modeled exciton energies and oscillator strengths represent an average of these parameters over the size of the probe spot. This inhomogeneity also results in additional broadening of the exciton and polariton spectral features, which is phenomenologically captured by the width parameter of the exciton oscillator. The dielectric function

for all other layers in the cavity structure are assumed to have no imaginary (absorptive) component. The pump-induced change in R is modeled by allowing the exciton oscillator to shift in energy, which causes the UP and LP features to shift and change in amplitude. The difference between these spectra, normalized to the unperturbed R spectrum is used to fit the $\Delta R/R$ data.

Determining the equilibrium oscillator parameters for the neutral exciton turns out to be non-trivial in our measurements. Ideally, a fit to the the polariton reflectance spectrum R measured during the pump-probe measurement would determine the equilibrium oscillator parameters, and only the center energy of that oscillator would be allowed to vary to fit the $\Delta R/R$ data. However, the R spectrum measured by the probe beam is too noisy to be used for reliable fitting in this manner [Supplementary Fig. 8(a)]. We are able to measure the R spectra of the polaritons separately using a white light source and a spectrometer, but due to the spatial inhomogeneity of TMD monolayers, the R spectrum is very sensitive to the location of the laser spot [Supplementary Fig. 8(b)]. Since these two measurements must be taken separately, this means that the R spectra measured with white light do not necessarily correspond to the $\Delta R/R$ spectra measured using the tunable probe. Since both the white light R spectrum and the R spectrum measured by the probe beam cannot be used to determine the equilibrium oscillator parameters, an alternative approach is used.

Instead of using a direct fit to the R spectrum, the unperturbed oscillator parameters are determined by leaving these parameters free when fitting the $\Delta R/R$ spectra in the minimally-detuned regime. For the initial fits to the $\Delta R/R$ spectra at 200 K, the four fitting parameters are equilibrium oscillator strength, width, energy, and the pump-induced shift ΔE. For a pump fluence of 1.47 GW/cm^2, the $\Delta R/R$ data is well-fit by a 161 µeV exciton shift, an oscillator strength $f_0 = 0.4$, with center energy $E_0 = 2.002$ eV and a damping coefficient $\gamma_0 = 40$ meV. These parameters yield an R spectrum [Supplementary Fig. 8(d)] that is within reasonable agreement with the measured reflectance spectra at 200 K [Supplementary Fig. 8(b)], establishing the consistency between our measured data and our fit parameters.

For the temperature-dependent $\Delta R/R$ spectra, a similar approach is used to model the data. Again, ideally the exciton oscillator parameters could be extracted from the reflectance spectra measured by the white light at each temperature, then seeded in the fit to the $\Delta R/R$ spectra to find the magnitude of the Stark shift. However, due to spatial inhomogeneity

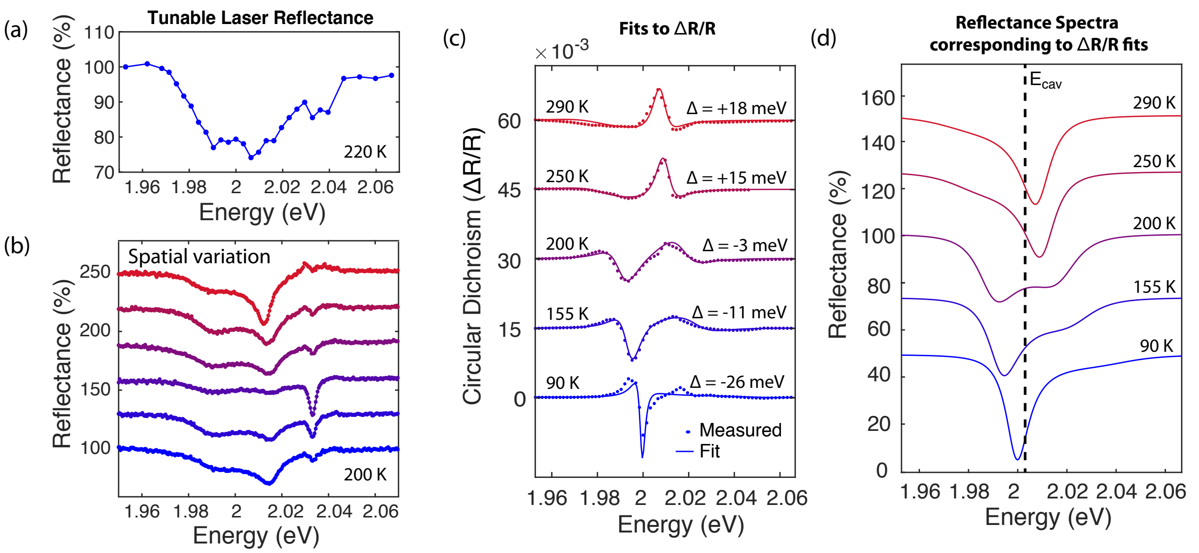

Supplementary Fig. 8. (a) Reflectance spectrum of polariton doublet measured with tunable laser, monochromator, and photodiode. (b) Reflectance spectra of polariton doublet at different sample locations measured with white light source and spectrometer showing variation in the relative weight of LP and UP. (c) Fits to $\Delta R/R$ spectra allowing exciton center energy, width, and oscillator strength to vary freely. (d) Reflectance spectra corresponding to the fit parameters found in (c).

in the sample, it is difficult to precisely correlate the reflectance spectrum to the $\Delta R/R$ spectrum at different temperatures. Because the oscillator parameters cannot be measured directly, in Fig. 3(b) of the main manuscript we model the exciton oscillator using the best-fit parameters found from the fits at 200 K, and model the shift of the exciton center energy at different temperatures using the slope of a linear fit to the temperature-dependent PL of WS_2 shown in Supplementary Fig. 1. This approach also accounts for the $\approx 10\%$ variation in the magnitude of the Stark shift at different temperatures due to differences pump-exciton detuning [Supplementary Fig. 5]. This extremely simplified model describes the salient features of the measured data, demonstrating how the change in the exciton center energy with temperature is the dominant cause of the changes in the observed spectral features with temperature.

The model agreement can be further improved if we account for the uncertainty in the exciton oscillator parameters by fitting the Stark shift spectra while allowing the oscillator width and center energy to vary within a reasonable amount at different temperatures. This fitting approach is used in Supplementary Fig. 8(c), where the magnitude of the Stark

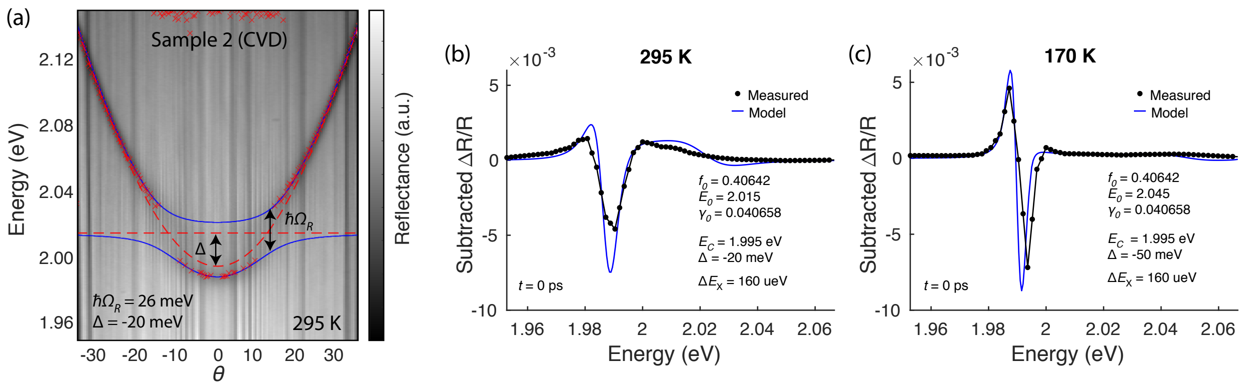

Supplementary Fig. 9. Analysis of highly-detuned polaritons (a) Momentum-space reflectance spectrum of Sample 2 at 295 K with fit to a Jaynes-Cummings model. (b,c) Cross section of Stark shift induced $\Delta R/R$ spectra shown in Fig. 4a of the main paper. The expected $\Delta R/R$ spectra for a 161 μeV exciton shift are calculated with the transfer-matrix model, using the same exciton oscillator strength and width as the modeled data in Figures 2 and 3.

shift is constrained to the 161 μeV value found from the fit at 200 K, and the exciton center energy and linewidth are allowed to vary with temperature. The Δ values found from these free fits remain consistent with the expected temperature-dependent shift of the WS_2 A exciton (Supplementary Fig. 1). Compared to the modeled data in the Fig. 3(b) that hold the exciton oscillator parameters constant with temperature, the improved agreement when using free fit parameters highlights how allowing for temperature-dependent oscillator parameters in fitting can further improve agreement with the model compared to the straightforward approach of the main text. The simulated R spectra corresponding to the best-fit parameters from these fits to the $\Delta R/R$ spectra are shown in Supplementary Fig. 8(d), which qualitatively agree with the measured spectra shown in Fig. 3(a) of the main paper. The agreement demonstrates how this minimally-constrained fitting procedure still captures the temperature dependence of both the $\Delta R/R$ spectra and the R spectra.

Analysis of Highly-detuned Polaritons

In Fig. 4 of the main text, we show the persistence of a pump-induced Stark shift signal with large polarization contrast into the highly-detuned polariton regime. The -20 meV detuning value at 295 K is calculated using a Jaynes-Cummings fit to the momentum-space re-

flectance spectrum of the sample, shown in Supplementary Fig. 9(a). Assuming a $\sim$ 30 meV shift in the exciton energy from 295 K to 170 K (as observed in the temperature-dependent PL spectra in Supplementary Fig. 1(c)), the cavity-exciton detuning is approximately -50 meV at 170 K.

To ensure that the measured $\Delta R/R$ signal agrees with our expectation, we use the transfer-matrix model to calculate the expected $\Delta R/R$ spectrum for these highly-detuned polaritons using reasonable estimations of the exciton oscillator parameters and the magnitude of the Stark shift. For these narrower spectral responses, we include a gaussian spectral averaging in the modeled data that accounts for the smearing caused by the bandwidth of the probe pulse ($\sim$ 3 meV). At 295 K the pump-exciton detuning is 525 meV, and at 170 K the pump-exciton detuning increases to 555 meV due to the 30 meV exciton shift. Both values are comparable to the $\sim$ 500 meV pump-exciton detuning in Figures 2 and 3 of the main paper. Since pump detuning and pump intensity are similar between the measurement in Fig. 4 and the measurements in Fig. 2 and 3 of the main paper, we expect a similar magnitude of the exciton Stark shift $\Delta E \approx 160$ μeV. The expected $\Delta R/R$ spectra for a 160 μeV exciton shift are calculated using the transfer-matrix model, shown in Supplementary Fig. 9(b,c). For this calculation, we use the same exciton oscillator strength and width values that were used for the modeled data in Figures 2 and 3. We observe good agreement between the measured $\Delta R/R$ spectra and the calculated $\Delta R/R$ spectra at 295 K and 170 K using these estimations, suggesting that the magnitude of the exciton shift is near the expected value of $\sim$ 160 μeV.

Uncertainty in Fluence-Dependent Fits

The fluence-dependent $\Delta R/R$ spectra are measured at four different pump powers spanning an order of magnitude [Fig. 2(b)]. Each of the four $\Delta R/R$ spectra are first fit using the four-free-parameter fitting procedure described above without constraining any of the four fitting parameters. The only difference between all of these measurements was the power of the pump pulse. Accordingly, a robust fit should show little variation between the oscillator parameters f_0, E_0, and γ_0 found from the different $\Delta R/R$ spectra. Instead, the differences in the magnitude of the $\Delta R/R$ signal should be captured by an increase in the shift of the oscillator ΔE. The best-fit parameters found from the unconstrained fit are shown in

Pump Fluence (GW/cm^2)	E_0 (eV)	f_0	γ_0 (eV)	ΔE (µeV)
0.141	2.0035	0.387	0.036	11
0.283	2.0043	0.390	0.043	26
0.735	2.0052	0.406	0.041	69
1.474	2.0058	0.442	0.042	172
**Std. Dev.**	**0.05 %**	**6.3 %**	**7.6 %**	

Supplementary Table I. Oscillator parameters found from unconstrained fit to 200 K $\Delta R/R$ spectra at different pump fluence. The extracted shift ΔE changes by an order of magnitude with increasing fluence, while the equilibrium oscillator parameters exhibit minimal variation.

Table I. f_0, E_0, and γ_0 only vary within $< 8\%$, while ΔE varies by an order of magnitude between the lowest pump fluence and the largest, establishing how our model is primarily sensitive to the magnitude of the Stark shift ΔE.

For an unconstrained fit with four free parameters, the interplay between different free parameters can lead to additional uncertainty in the extracted values. To understand the impact of these kinds of effects on the shift magnitude ΔE, we re-fit the $\Delta R/R$ spectrum at each fluence using a more constrained fit where only ΔE was allowed to vary. The associated fitting uncertainty in ΔE is determined by fitting the $\Delta R/R$ spectrum at each fluence with this one-parameter fit four different times; each time using one set of the f_0, E_0, and γ_0 values found from the unconstrained fit at other fluences. We found a standard deviation in the extracted ΔE at each fluence to be 7-8%, which we take as a reasonable estimate of the uncertainty for ΔE arising from the fitting procedure.

Model performance at low temperatures

At the lower end of the temperature ranges we measured, particularly at 90 K, the oscillator strength of the charged exciton increases [7] and may become non-negligible. This may explain why the reflectance spectrum at 90 K still exhibits a doublet feature [Fig. 3(b)], since at this temperature the charged exciton comes into resonance with the cavity. Our simple model does not include an oscillator for the charged exciton, so the Stark-induced

$\Delta R/R$ feature near this second resonance is not captured by our fit at 90 K [Fig. 3(b)].

Additionally, for most of the measured temperatures, the width of the polariton resonances are larger than the smearing caused by the bandwidth of the probe pulse (~ 3 meV). At 90 K, where the exciton resonance narrows significantly and the LP branch becomes more photonic, this is no longer the case. This likely explains why our model anticipates a sharper feature in the $\Delta R/R$ signal than measured: each data point in the spectral measurement is averageing over ~ 3 meV, which smears out our signal and this smearing is not captured by our model.

---

* n-stern@northwestern.edu

[1] Xiaoze Liu, Wei Bao, Quanwei Li, Chad Ropp, Yuan Wang, and Xiang Zhang, "Control of coherently coupled exciton polaritons in monolayer tungsten disulphide," Phys. Rev. Lett. **119**, 027403 (2017).

[2] Lei Wang, I Meric, PY Huang, Q Gao, Y Gao, H Tran, T Taniguchi, Kenji Watanabe, LM Campos, DA Muller, *et al.*, "One-dimensional electrical contact to a two-dimensional material," Science **342**, 614–617 (2013).

[3] JP Sokoloff, M Joffre, B Fluegel, D Hulin, M Lindberg, Stephan W Koch, A Migus, A Antonetti, and N Peyghambarian, "Transient oscillations in the vicinity of excitons and in the band of semiconductors," Physical Review B **38**, 7615 (1988).

[4] Christoph Lange, Emiliano Cancellieri, Dmitry Panna, David M Whittaker, Mark Steger, David W Snoke, Loren N Pfeiffer, Kenneth W West, and Alex Hayat, "Ultrafast control of strong light–matter coupling," New Journal of Physics **20**, 013032 (2018).

[5] Alex Hayat, Christoph Lange, Lee A Rozema, Ardavan Darabi, Henry M van Driel, Aephraim M Steinberg, Bryan Nelsen, David W Snoke, Loren N Pfeiffer, and Kenneth W West, "Dynamic Stark effect in strongly coupled microcavity exciton polaritons," Phys. Rev. Lett. **109**, 033605 (2012).

[6] JJ Hopfield, "Theory of the contribution of excitons to the complex dielectric constant of crystals," Phys. Rev. **112**, 1555 (1958).

[7] Ashish Arora, Thorsten Deilmann, Till Reichenauer, Johannes Kern, Steffen Michaelis de Vasconcellos, Michael Rohlfing, and Rudolf Bratschitsch, "Excited-state trions in monolayer WS_2,"

Physical Review Letters **123**, 167401 (2019).
